# C-X-C motif chemokine ligand 10 produced by mouse Sertoli cells in response to mumps virus infection induces male germ cell apoptosis

**DOI:** 10.1038/cddis.2017.560

**Published:** 2017-10-26

**Authors:** Qian Jiang, Fei Wang, Lili Shi, Xiang Zhao, Maolei Gong, Weihua Liu, Chengyi Song, Qihan Li, Yongmei Chen, Han Wu, Daishu Han

**Affiliations:** 1 Department of Cell Biology, Institute of Basic Medical Sciences, Chinese Academy of Medical Sciences, School of Basic Medicine, Peking Union Medical College, Beijing, China; 2 Joint International Research Laboratory of Agriculture and Agri-product Safety, Institute of Epigenetics and Epigenomics, College of Animal Science and Technology, Yangzhou University, Yangzhou, China; 3 Institute of Medical Biology, Chinese Academy of Medical Sciences, Kunming, China

## Abstract

Mumps virus (MuV) infection usually results in germ cell degeneration in the testis, which is an etiological factor for male infertility. However, the mechanisms by which MuV infection damages male germ cells remain unclear. The present study showed that C-X-C motif chemokine ligand 10 (CXCL10) is produced by mouse Sertoli cells in response to MuV infection, which induces germ cell apoptosis through the activation of caspase-3. CXC chemokine receptor 3 (CXCR3), a functional receptor of CXCL10, is constitutively expressed in male germ cells. Neutralizing antibodies against CXCR3 and an inhibitor of caspase-3 activation significantly inhibited CXCL10-induced male germ cell apoptosis. Furthermore, the tumor necrosis factor-*α* (TNF*-α*) upregulated CXCL10 production in Sertoli cells after MuV infection. The knockout of either CXCL10 or TNF-*α* reduced germ cell apoptosis in the co-cultures of germ cells and Sertoli cells in response to MuV infection. Local injection of MuV into the testes of mice confirmed the involvement of CXCL10 in germ cell apoptosis *in vivo*. These results provide novel insights into MuV-induced germ cell apoptosis in the testis.

Mumps is a contagious disease caused by the mumps virus (MuV) and is characterized by painful parotitis. Orchitis is the most common extra-parotid gland complication of mumps that affects up to 30% of mumps cases in post-pubertal men.^1^ More than 50% of patients with bilateral mumps orchitis experience infertility.^2^ Moreover, a major pathological manifestation of mumps orchitis is germ cell degeneration.^3^ Mumps orchitis is associated with the presence of MuV in the testis, thereby suggesting that MuV should directly induce pathogenesis.^4^ However, the mechanisms underlying MuV-induced male germ cell degeneration remain unknown.

Spermatogenesis and steroidogenesis are two major functions of the testis. Several inflammatory cytokines are involved in testis pathophysiology.^5^ Interleukin 1 (IL-1), IL-6 and tumor necrosis factor-*α* (TNF-*α*) have important roles in regulating spermatogenesis under physiological conditions.^6^ However, these cytokines can be upregulated and impair testicular functions under inflammatory conditions.^7^ High levels of IL-1, IL-6 and TNF-*α* inhibit steroidogenesis in Leydig cells.^8, 9, 10^ Moreover, TNF-*α* upregulation induced male germ cells apoptosis in an experimental autoimmune orchitis model.^11^ We recently demonstrated that the C-X-C motif chemokine ligand 10 (CXCL10) expression is remarkably upregulated in Leydig and Sertoli cells in response to MuV infection,^12^ but the effect of the increased CXCL10 on testicular function remains unknown.

CXCL10 was initially identified as an IFN-*γ*-inducible cytokine,^13^ and functions by binding to CXC chemokine receptor 3 (CXCR3).^14^ CXCL10 is a pleiotropic cytokine capable of exerting various functions, including chemotactic homing of leukocytes, induction of cell apoptosis and regulation of cell proliferation.^15^ Moreover, CXCL10 is involved in the pathogenesis of various autoimmune and infectious diseases.^16, 17^ Notably, CXCL10 upregulation in simian immunodeficiency virus (SIV) encephalitis induces neuronal apoptosis.^18^ The downregulation of CXCR3 expression reduced neuronal apoptosis in a mouse model of West Nile virus encephalitis.^19^ Increased CXCL10 level in the cerebrospinal fluid of individuals infected with human immunodeficiency virus (HIV) has been associated with the progression of neuropsychiatric impairment.^20^ These studies indicated that CXCL10 upregulation is involved in the pathogenesis of viral encephalitis.

CXCL10 is expressed in rat Leydig cells and can be upregulated by TNF-*α* and IFN-*γ*.^21^ Sendai viral infection induces CXCL10 expression in rat testicular somatic cells, including testicular macrophages, Sertoli, Leydig and peritubular myoid cells.^22^ By contrast, the Sendai virus does not induce CXCL10 expression in rat male germ cells. We recently demonstrated that MuV dramatically induces CXCL10 expression in mouse Leydig and Sertoli cells but not in germ cells.^12^ We speculated that CXCL10 production by testicular somatic cells in response to viral infections might be detrimental to male germ cells. This study examined the role of MuV-induced CXCL10 production by mouse Sertoli cells in inducing male germ cell apoptosis.

## Results

### Expression of CXCR3 in testicular cells

**Figure 1:**
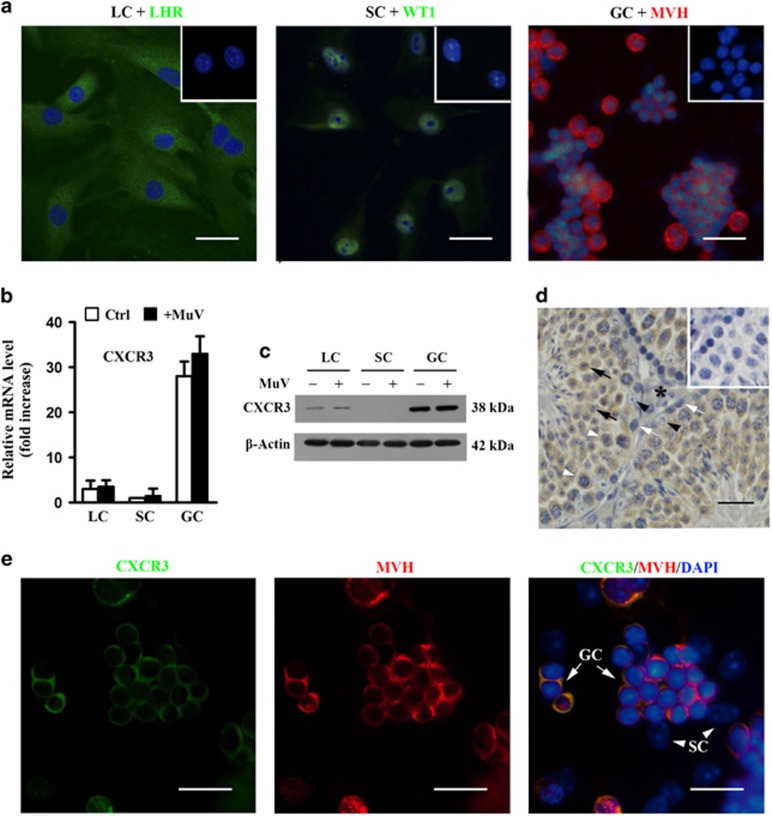
Expression of CXCR3 in mouse testicular cells. (**a**) Identification of testicular cells. Major testicular cells, including Leydig cells (LC), Sertoli cells (SC) and germ cells (GC), were isolated from 4-week-old C57BL/6J mice. Each cell type was identified by immunostaining for respective markers: LHR for LC, WT1 for SC and MVH for GC. Insets in the upper right corners of the images represent negative controls, in which the preimmune rabbit sera were used as the first antibodies. (**b**) CXCR3 mRNA. Cells were cultured in the absence (Ctrl) and presence of 10^7^ PFU/ml MuV for 24 h. Total RNA was extracted from the testicular cells, and relative mRNA level of CXCR3 was determined using real-time qRT-PCR by normalizing to *β*-Actin. The lowest CXCR3 mRNA level in SC was set as ‘1’. The fold increases in LC and GC compared to SC were presented. (**c**) CXCR3 protein. Testicular cell lysates were subjected to western blot analysis to probe CXCR3 using specific antibodies. *β*-Actin was probed as loading controls. (**d**) CXCR3 distribution in the testis. Immunohistochemistry was performed to localize CXCR3 on the paraffin sections of the testis from 5-week-old C57BL/6J mice. The inset in the upper right corner of the image in the right panel represents negative control, in which the preimmune goat serum was used as the first antibody. Black arrows, black arrowheads, white arrows, white arrowheads and asterisk indicate round spermatids, SC, spermatogonia, spermatocytes and interstitial cells, respectively. (**e**) CXCR3 locations in GC and SC co-cultures. IF co-staining with CXCR3 (green) and MVH (red) for GC and SC co-cultures. Scale bar=20 *μ*m. Images are the representatives of at least three experiments. Real-time qRT-PCR data are the means±S.E.M. of three experiments

CXCL10 is significantly produced by mouse Leydig and Sertoli cells in response to MuV infection.^12^ To reveal the potential role of MuV-induced CXCL10 in the testis, we examined CXCR3 expression in major testicular cells. Leydig, Sertoli and germ cells were identified by staining with luteinizing hormone receptor (LHR), Wilms tumor nuclear protein 1 (WT1) and mouse VASA homolog (MVH), respectively (Figure 1a). The purity of each cell types was >95%. Real-time quantitative RT-PCR (qRT-PCR) results showed that the CXCR3 mRNA level was considerably higher in male germ cells than in Leydig and Sertoli cells (Figure 1b). MuV infection did not significantly affect CXCR3 expression in testicular cells. Western blot analysis demonstrated that CXCR3 protein was abundantly detected in germ cells in the absence and presence of MuV (Figure 1c). Moreover, CXCR3 protein was faintly detected in Leydig cells. By contrast, CXCR3 protein was not detected in Sertoli cells. Immunohistochemistry (Figure 1d) and immunofluorescence (IF) co-staining of CXCR3 and MVH (Figure 1e) confirmed that CXCR3 protein was obviously located in male germ cells. A weak CXCR3 signal was observed in interstitial cells (asterisk) (Figure 1d), but not detected in Sertoli cells (Figure 1e).

### CXCL10-induced germ cell apoptosis

**Figure 2:**
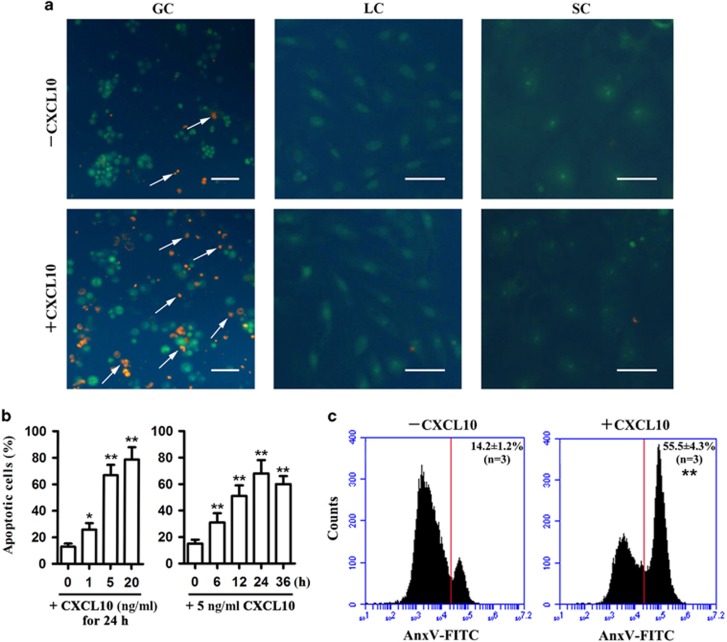
Germ cell apoptosis. (**a**) AO/EB staining. Testicular cells, including GC, LC and SC, were isolated from 4-week-old mice and cultured *in vitro* in the absence of CXCL10 (upper panels) or presence of 5 ng/ml CXCL10 (lower panels) for 24 h. AO/EB solution was added to cultures at a dilution of 1:1000. After 1 min, apoptotic cells were stained as ‘orange’ (arrows) and living cells were stained as ‘green.’ (**b**) Dose- and time-dependent effects of CXCL10 on germ cell apoptosis. Germ cells were cultured in the presence of the indicated doses of CXCL10 for 24 h (left panel) or in the presence of 5 ng/ml CXCL10 for specific durations (right panel). Percentages of apoptotic cells were calculated based on AO/EB staining results. At least 500 cells were spontaneously counted. (**c**) Flow cytometry. Germ cells were cultured in the presence of 5 ng/ml CXCL10 for 24 h. Cells were labeled with Annexin V (AnxV)-FITC for 15 min and analyzed using BD Accuri C6 flow cytometer. Images are the representatives of at least three independent experiments, scale bar=20 *μ*m. Data are the means±S.E.M. of three experiments. *P<0.05 and **P<0.01

Considering that CXCL10 induces neuronal apoptosis,^18^ we examined the apoptosis of testicular cells in the presence of recombinant mouse CXCL10. Acridine orange/ethidium bromide (AO/EB) staining results showed that apoptotic male germ cells (arrows) were significantly increased at 24 h in the presence of 5 ng/ml CXCL10 (Figure 2a, lower left panel). However, certain apoptotic germ cells were also observed in control cells in the absence of CXCL10 (Figure 2a, upper left panel), suggesting that the male germ cells underwent spontaneous apoptosis during culture *in vitro*. By contrast, CXCL10 did not induce the apoptosis of Leydig (Figure 2a, middle panels) or Sertoli cells (right panels). The dose-dependent (Figure 2b, left panel) and time-dependent (right panel) effects of CXCL10 on male germ cell apoptosis were quantitatively analyzed. Furthermore, flow cytometry analysis confirmed that apoptotic germ cells were significantly increased at 24 h in the presence of 5 ng/ml CXCL10 (Figure 2c). Notably, both AO/EB staining and flow cytometry analysis showed comparable results, indicating that these two approaches are reliable in determining germ cell apoptosis.

### Activation of caspase-3 in germ cell apoptosis

**Figure 3:**
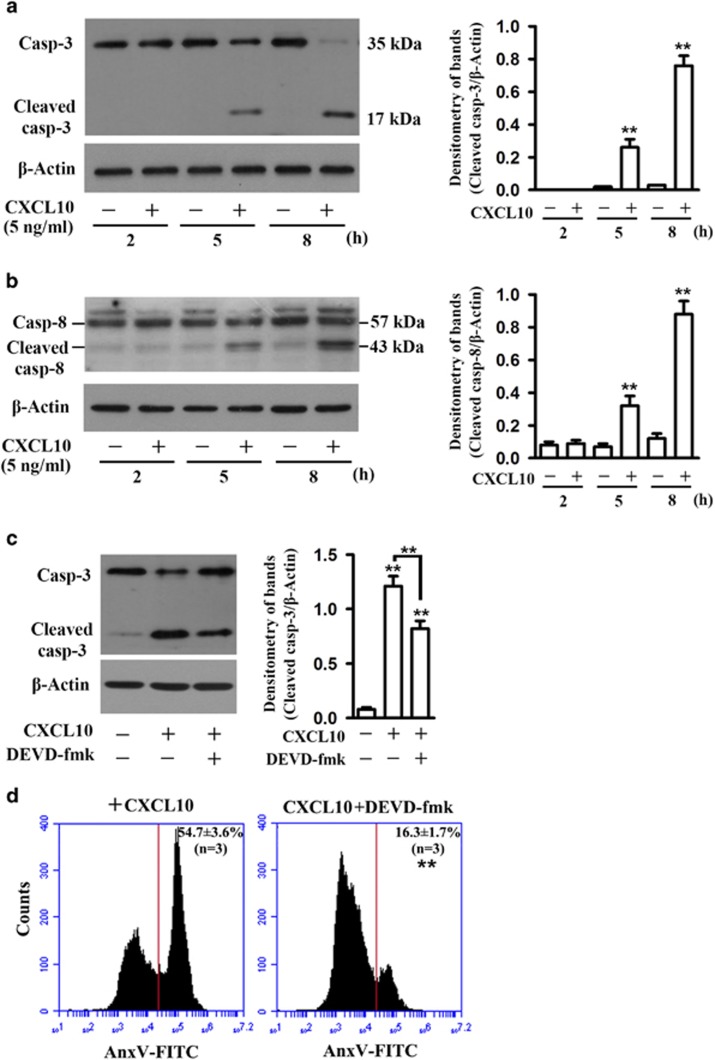
Caspase activation. (**a**) Caspase 3 activation. Germ cells were cultured in the absence (−) and presence (+) of 5 ng/ml CXCL10 for the specific durations. The full lengths (35 kDa) of caspase 3 (Casp-3) and cleaved Casp-3 (17 kDa) in cell lysates were determined by western blot analysis. *β*-Actin was used as loading controls. Signal densities were quantified by densitometry (right panel). (**b**) Caspase 8 activation. Germ cells were treated as described in (**a**), full length and cleavage of Casp-8 were determined by western blot analysis. (**c**) Inhibition of Casp-3 activation. Germ cells were treated with CXCL10 or with CXCL10 in the presence of 10 *μ*M DEVD-fmk, an inhibitor of Casp-3 activation, for 8 h. Casp-3 was determined by western blot analysis. (**d**) Germ cell apoptosis. Germ cells were cultured in the presence of CXCL10 along (left panel) or in the presence of CXCL10 and DEVD-fmk (right panel) for 24 h. Apoptotic cells were labeled with AnxV-FITC and analyzed using flow cytometry. Images are the representatives of at least three independent experiments. Data of flow cytometry are the means±S.E.M. of three experiments. **P<0.01

To further understand the mechanism by which CXCL10 induced germ cell apoptosis, we examined the activation of the caspase cascade, an important apoptotic pathway.^23^ CXCL10 induced caspase-3 activation in germ cells in a time-dependent manner (Figure 3a). The cleavage of caspase-3 was remarkable at 5 and 8 h after the presence of CXCL10 (Figure 3a, left panel). Band intensity was quantified by densitometry (Figure 3a, right panel). Caspase-8 cleavage is essential to activate caspase-3. We found that caspase-8 was significantly cleaved in germ cells by CXCL10 (Figure 3b). DEVD-fmk, an inhibitor of caspase-3 activation, efficiently inhibited caspase-3 cleavage at 8 h in the presence of CXCL10 (Figure 3c). Accordingly, DEVD-fmk significantly reduced apoptotic germ cell numbers 24 h after the presence of CXCL10 (Figure 3d).

### MuV-induced apoptosis of male germ cells co-cultured with Sertoli cells

**Figure 4:**
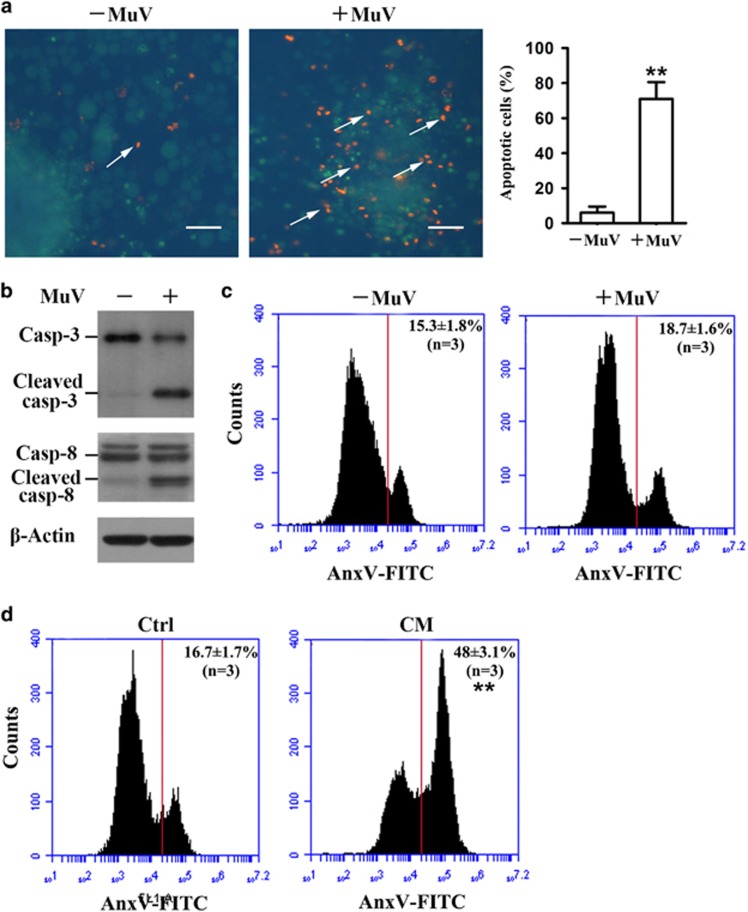
MuV-induced male germ cell apoptosis. (**a**) MuV-induced apoptosis of male germ cells co-cultured with Sertoli cells. Sertoli and germ cells were isolated from 4-week-old mice and co-cultured at a ratio of 1:5 for 24 h. The co-cultures were infected with 1 × 10^7^ PFU/ml MuV (middle panel). Cells without MuV infection served as controls (left panel). Apoptotic cells (arrows) were determined using AO/EB staining at 24 h after MuV infection. Percentages of apoptotic germ cells were calculated based on AO/EB staining (right panel). At least 500 cells were spontaneously counted. (**b**) Caspase activation. The co-cultures of Sertoli and germ cells were infected as described in **a**. Germ cells were collected by treatment with hypotonic solution (20 mM Tris-HCl, pH 7.4) for 1 min. Cell lysates were subject to western blot analysis to probe caspases 3 and 8. (**c**) Apoptosis of male germ cells cultured alone. Male germ cells of 4-week-old mice were cultured in the absence (left panel) and presence (right panel) of MuV for 24 h. Apoptotic germ cells were determined using flow cytometry after labeling cells with AnxV-FITC. (**d**) Apoptosis of male germ cells in the conditional medium (CM). CM was collected by a centrifugation of the supernatant of Sertoli cells 24 h post MuV infection. Germ cells were cultured in CM for 24 h and apoptotic germ cells were analyzed by flow cytometry. The supernatant of Sertoli cells without MuV infection served as the control (ctrl). Images are the representatives of at least three experiments. Scale bar=20 *μ*m. Data are the means±S.E.M. of three experiments. **P<0.01

MuV induces CXCL10 production in Sertoli cells,^12^ thus, we speculated that CXCL10 produced by Sertoli cells might induce germ cell apoptosis in a paracrine fashion. Therefore, we analyzed germ cell apoptosis in the co-cultures of germ cells and Sertoli cells in response to MuV infection. AO/EB staining showed that MuV remarkably increased apoptotic germ cells (arrows) 24 h after infection (Figure 4a, middle panel). In controls, only a few apoptotic germ cells were observed in the co-cultures of germ cells and Sertoli cells without MuV infection (Figure 4a, left panel). Percentages of apoptotic germ cells were quantitatively analyzed based on AO/EB staining (Figure 4a, right panel). In accordance with germ cell apoptosis, MuV evidently induced the activation of caspase-3 and caspase-8 in the co-cultures (Figure 4b). By contrast, flow cytometry analysis showed that MuV did not significantly induce apoptosis of male germ cells cultured alone *in vitro* (Figure 4c). However, apoptotic germ cells were significantly increased in the conditional medium from Sertoli cells 24 h after MuV infection (Figure 4d).

### Role of CXCL10 in MuV-induced germ cell apoptosis

**Figure 5:**
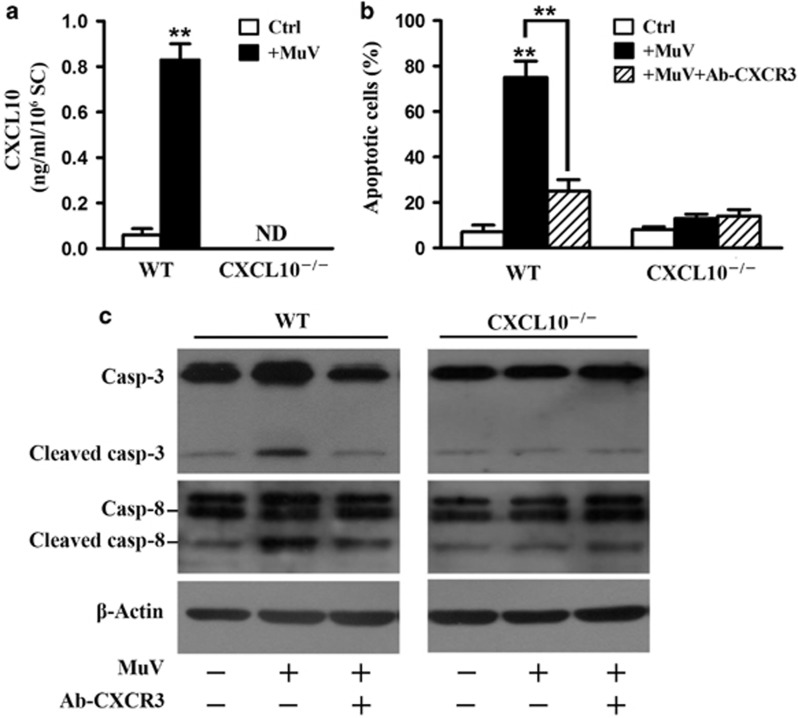
Role of CXCL10 in MuV-induced germ cell apoptosis. (**a**) MuV-induced CXCL10 production. Sertoli and germ cells from WT and CXCL10^−/−^ mice were co-cultured in the absence (Ctrl) or presence (+ MuV) of 10^7^ PFU/ml MuV. At 24 h after MuV infection, CXCL10 levels in media were measured using ELISA. (**b**) MuV-induced germ cell apoptosis. The co-cultures of Sertoli and germ cells were infected with MuV or with MuV in the presence of neutralizing antibodies against CXCR3 (Ab-CXCR3) for 24 h. Apoptotic cells were quantitatively analyzed based on AO/EB staining. (**c**) Caspase activation. Co-cultures were treated as described in (**b**). The activation of caspases 3 and 8 in germ cells was determined by western blot analysis. Images are the representatives of at least three experiments. Data are the means±S.E.M. of three experiments. **P<0.01

To examine the role of CXCL10 produced by Sertoli cells in MuV-induced germ cell apoptosis, we compared the apoptosis of germ cells co-cultured with Sertoli cells from wild-type (WT) and CXCL10^−/−^ mice. CXCL10 levels in the media of WT cells were significantly increased 24 h after MuV infection (Figure 5a). By contrast, CXCL10 was not detectable in the media of CXCL10^−/−^ cells. MuV significantly induced germ cell apoptosis in co-cultures of WT cells 24 h after infection (Figure 5b). Notably, neutralizing antibodies against CXCR3 (ab-CXCR3) significantly reduced MuV-induced germ cell apoptosis in co-cultures of WT cells. By contrast, MuV and ab-CXCR3 did not significantly affected germ cell apoptosis in CXCL10^−/−^ cells. Accordingly, MuV-induced cleavages of caspase-3 and caspase-8 were inhibited by ab-CXCR3 in WT germ cells (Figure 5c, left panels). MuV did not induce the cleavages of caspase-3 and caspase-8 in CXCL10^−/−^ germ cells (Figure 5c, right panels).

### TNF-*α*-induced CXCL10 production

**Figure 6:**
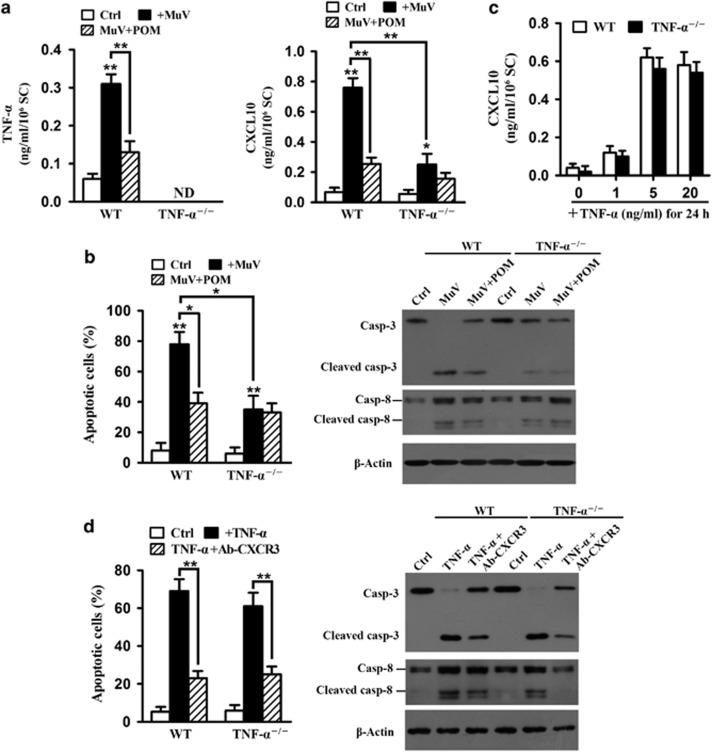
Role of TNF-*α* in inducing CXCL10 expression. (**a**) MuV-induced TNF-*α* and CXCL10 production. Sertoli and germ cells of 4-week-old WT or TNF-*α*^−/−^ mice were co-cultured and infected with MuV or with MuV in the presence of pomalidomide (POM), an inhibitor of TNF-*α* secretion for 24 h. Co-cultures without MuV infection served as controls (Ctrl). TNF-*α* (left panel) and CXCL10 (right panel) levels in the culture media were measured using ELISA. (**b**) MuV-induced germ cell apoptosis. Co-cultures were treated as described in **a**. Apoptotic germ cells were determined based on AO/EB staining and confirmed by determination of caspase cleavages by Western blot analysis. (**c**) Induction CXCL10 production by recombinant TNF-*α*. WT or TNF-*α*^−/−^ cells were co-cultured in the presence of the indicated doses of recombinant mouse TNF-*α* for 24 h. CXCL10 levels in media were determined using ELISA. (**d**) TNF-*α*-induced germ cells apoptosis. WT or TNF-*α*^−/−^ cell co-cultures were treated with 5 ng/ml TNF-*α* or with TNF-*α* in the presence of Ab-CXCR3 for 24 h. Apoptotic germ cells were determined based on AO/EB staining and caspase activation was assessed by western blot analysis. Data are the means±S.E.M. of three independent experiments. *P<0.05 and **P<0.01

Given that MuV infection upregulates TNF-*α* production in mouse Sertoli cells and TNF-*α* induces CXCL10 expression,^12, 21^ we examined the role of autocrine TNF-*α* in inducing CXCL10 expression in Sertoli cells after MuV infection. Enzyme-linked immunosorbent assay (ELISA) results confirmed that MuV infection significantly increased the TNF-*α* (Figure 6a, left panel) and CXCL10 (right panel) levels in the co-cultures of WT cells. An inhibitor of TNF-*α* secretion, pomalidomide,^24^ significantly reduced the TNF-*α* level. TNF-*α* was abolished in TNF-*α*^−/−^ cells (Figure 6a, left panel). MuV significantly increased CXCL10 levels in the media of both WT and TNF-*α*^−/−^ cells (Figure 6a, right panel); however, the CXCL10 level in TNF-*α*^−/−^ cells was significantly lower than that in WT cells in response to MuV infection. Notably, pomalidomide significantly inhibited MuV-induced CXCL10 production in WT cells but not in TNF-*α*^−/−^ cells. MuV significantly induced germ cell apoptosis and caspase activation in the co-cultures of WT cells (Figure 6b). By contrast, MuV did not significantly induce germ cell apoptosis in TNF-*α*^−/−^ cells. Recombinant mouse TNF-*α* induced CXCL10 production at comparable levels in Sertoli and germ cell co-cultures of WT and TNF-*α*^−/−^ mice in a dose-dependent manner (Figure 6c). Accordingly, TNF-*α* induced germ cell apoptosis and caspase activation in WT and TNF-*α*^−/−^ cell co-cultures, which were significantly reduced by ab-CXCR3 (Figure 6d). These results indicated that TNF-*α* upregulates CXCL10 production in Sertoli cells in an autocrine manner and CXCL10 induces germ cell apoptosis.

### MuV-induced CXCL10 production and germ cell apoptosis in the testis

**Figure 7:**
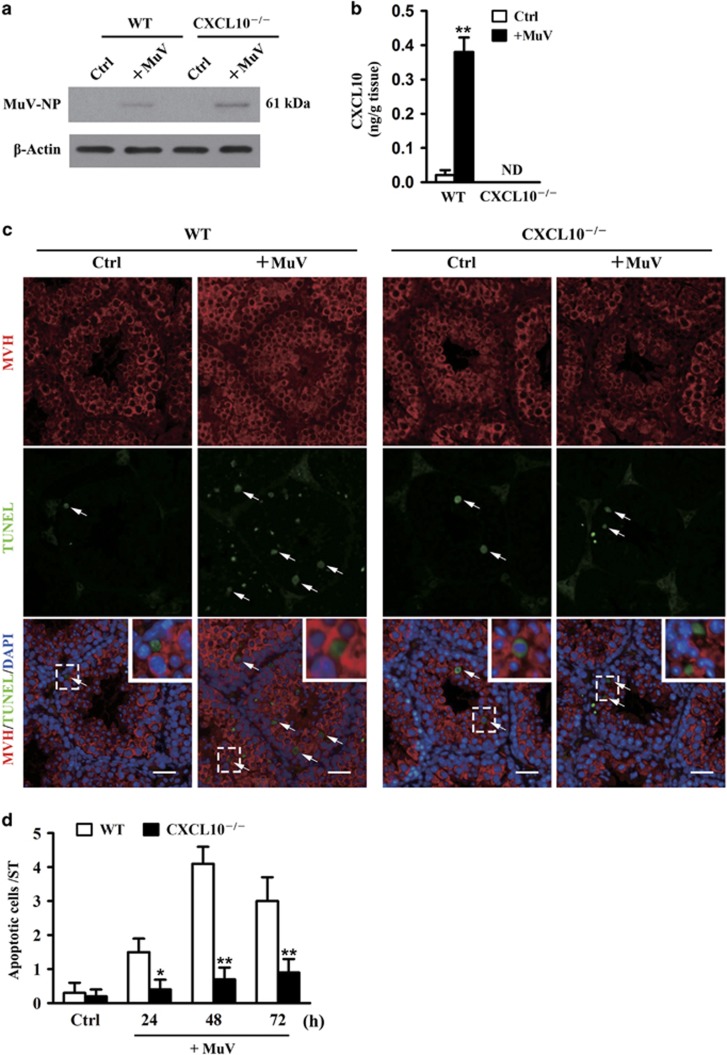
MuV-induced CXCL10 production and germ cell apoptosis in the testis. MuV (1 × 10^7^ PFU) in 10 *μ*l of PBS was injected into the testis of 5-week-old WT and CXCL10^−/−^ mice. The contralateral testis that was injected with an equal volume of PBS alone served as Ctrl. (**a**) MuV detection. MuV-NP in testicular lysates was detected by western blot analysis at 24 h after MuV injection. (**b**) CXCL10 level. The testis was lysed by grinding in PBS at 24 h after MuV injection. CXCL10 levels in the lysates were measured using ELISA. (**c**) Apoptosis of male germ cells. The testis of WT (left panels) and CXCL10^−/−^ (right panels) mice were injected with PBS or MuV. After 24 h, apoptotic germ cells in paraffin sections were detected using co-staining of TUNEL and IF with antibodies to MVH. Insets in upper right corners represent the higher resolution for dotted box areas (lower panels). (**d**) A time-dependent germ cell apoptosis. The testes of WT and CXCL10^−/−^ mice were injected with MuV for the indicated durations. Apoptotic germ cells were quantitatively analyzed based on the co-staining of TUNEL and IF. Apoptotic germ cell numbers per tubular section were presented. One hundred tubules per testis were counted. Images are the representatives of three mice. Scale bar=40 *μ*m. Data represent the means±S.E.M. of three mice. *P<0.05 and **P<0.01

To examine the involvement of CXCL10 in MuV-induced germ cell apoptosis *in vivo*, the testes of 5-week-old WT and CXCL10^−/−^ mice were locally injected with 1 × 10^7^ plaque forming unit (PFU) MuV in 10 *μ*l PBS. MuV nucleoprotein (MuV-NP) was detected in the testes of both WT and CXCL10^−/−^ mice 24 h after MuV injection (Figure 7a). In the controls, MuV-NP was not detected in the testes that were injected with PBS alone. ELISA results showed that the CXCL10 level was dramatically increased in testicular lysates of WT mice 24 h after MuV injection (Figure 7b). CXCL10 was not detected in the testes of CXCL10^−/−^ mice. Co-staining results demonstrated that MuV injection significantly increased apoptotic germ cells (arrows) in WT mice after 48 h, while only a few apoptotic germ cells were observed in the testes of control WT mice (Figure 7c, left panels). By contrast, apoptotic germ cells were not increased in CXCL10^−/−^ mice 48 h post MuV injection (Figure 7c, left panels). Apoptotic germ cell numbers per tubular section were quantitatively analyzed based on Terminal deoxynucleotidyl transferase-mediated dUTP nick end labeling (TUNEL) assay (Figure 7d). These results suggested that the increased CXCL10 level in the testis is associated with male germ cell apoptosis in response to MuV injection.

### Effect of MuV on spermatogenesis

**Figure 8:**
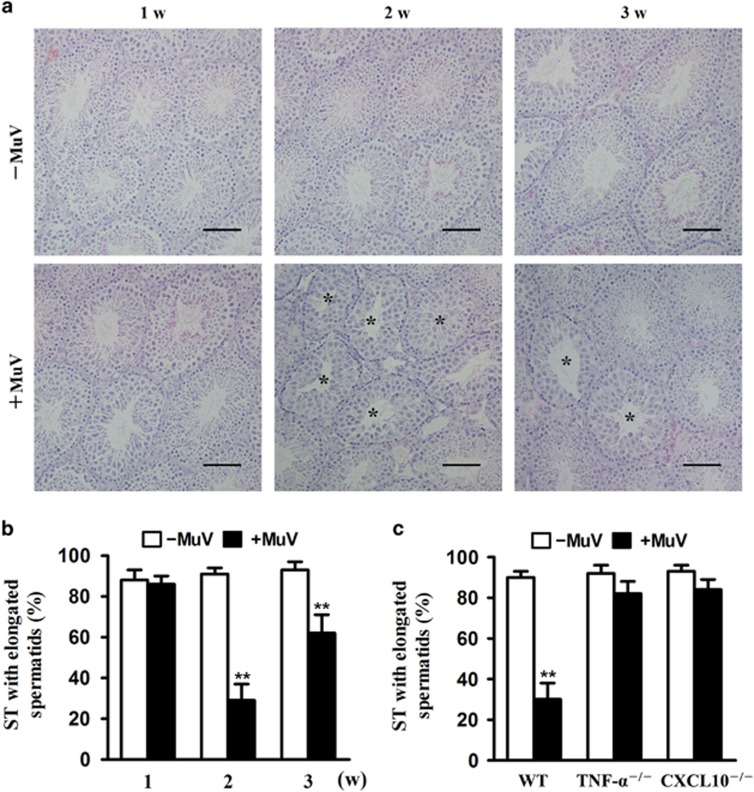
MuV-mediated impairment of spermatogenesis. (**a**) Histological analysis. MuV (1 × 10^7^ PFU) in 10 *μ*l of PBS was injected into the testis of 5-week-old C57BL/J6 mice (lower panels). Equal volume of PBS alone was injected into the contralateral testis for the control (upper panels). Histological analysis was performed on the paraffin sections after hematoxylin and eosin (HE) staining. Asterisk indicate seminiferous tubules (ST) without elongated spermatids. (**b**) Quantification of spermatogenesis impairment. C57BL/J6 mice were treated as described in **a**. The ratio of the ST with elongated spermatids in lumen was determined based on histological analysis. A total of 200 tubules in three sections were spontaneously counted for spermatogenesis examination. (**c**) Spermatogenesis impairment in TNF-*α*^−/−^ and CXCL10^−/−^ mice. 5-week-old TNF-*α*^−/−^ and CXCL10^−/−^, as well as respective control mice, were treated as described in (**a**). The ratio of ST with elongated spermatids in lumen was determined at 2 weeks after MuV injection. Images are the representatives of three mice. Scale bar=100 *μ*m. Data represent the means±S.E.M. of three mice. **P<0.01

To evaluate pathological consequences of MuV infection in male infertility/subfertility, we examined the spermatogenesis status. Local injection of MuV remarkably reduced the seminiferous tubules with elongated spermatids in the lumen at 2 weeks after injection (Figure 8a, lower panels). However, the impaired spermatogenesis was observed 1 and 3 weeks after MuV injection. The spermatogenesis status was quantitatively analyzed (Figure 8b). Further, we examined the roles of TNF-*α* and CXCL10 in the MuV-mediated impairment of spermatogenesis. We showed that either mutation of TNF-*α* or CXCL10 significantly increased the seminiferous tubules with elongated spermatids at 2 weeks after MuV injection (Figure 8c).

## Discussion

MuV infection usually impairs spermatogenesis, but the underlying mechanisms remain to be clarified. This study demonstrated that the CXCL10 produced by Sertoli cells in response to MuV infection induces germ cell apoptosis and TNF-*α* upregulates CXCL10 production in an autocrine manner. These results provide novel insights into the mechanisms underlying MuV-impaired spermatogenesis.

We recently found that mouse Leydig and Sertoli cells remarkably produced CXCL10 in response to MuV infection.^12^ To determine the potential role of CXCL10 in the MuV-infected testes, we examined the CXCR3 distribution in testicular cells. CXCR3 was predominantly expressed in male germ cells, suggesting that CXCL10 acts on germ cells. Moreover, CXCL10 induced germ cell apoptosis *in vitro*. Flow cytometry is a common approach for quantitatively analyzing the apoptosis of suspended cells after labeling with Annexin V-FITC. In the present study, the apoptosis of male germ cells cultured alone was analyzed by flow cytometry. However, we determined germ cell apoptosis using AO/EB staining in co-cultures of Sertoli and germ cells, because the germ cells firmly bound to Sertoli cells and could not be collected for flow cytometry. AO/EB staining approach has been used to detect cell apoptosis.^25^ We found that both flow cytometry and AO/EB staining gave comparable results, confirming that these two approaches are consistent for measuring male germ cell apoptosis.

To further understand the mechanisms underlying CXCL10-induced male germ cell apoptosis, we examined the activation of caspase-3 in male germ cells. Caspase-3 activation, which is induced by caspase-8 activation, is a critical pathway in executing cell apoptosis.^26^ We demonstrated that CXCL10 activated caspase-3 and caspase-8 in male germ cells. Notably, DEVD-fmk, a caspase-3 inhibitor,^27^ protected germ cells from CXCL10-induced apoptosis. These observations suggested that CXCL10 induces germ cell apoptosis via the activation of caspase cascades. In accordance with the *in vitro* results, we confirmed the association between CXCL10 upregulation and germ cell apoptosis in the testes after MuV infection *in vivo*. We recently found that MuV infection suppresses testosterone synthesis in mouse Leydig cells.^12^ However, whether the inhibition of testosterone synthesis contributes to MuV-impaired spermatogenesis remains to be clarified.

We further analyzed spermatogenesis status in response to MuV injection. The ratio of the seminiferous tubules containing elongated spermatids was significantly reduced at 2 weeks after local MuV injection in WT mice; however, this phenotype was recovered at 3 weeks. We did not observe permanent impairment of spermatogenesis *in vivo* after MuV infection. These results agreed with the previous observations that mice are resistant to MuV infection.^28^ Several aspects may be responsible for the resistance to MuV in mice: (1) mice adopt efficient antiviral ability. A previous study showed that murine Leydig cells exhibit higher efficient antiviral response than their human counterparts.^29^ Accordingly, various viral infections lead to orchitis in human beings, but natural viral orchitis has not been observed in murine animals.^30^ (2) MuV does not efficiently proliferate in mice after infection *in vivo*. MuV was significantly removed from the testis several days after local injection (data not shown). Therefore, MuV only transiently affects the mouse testis. (3) Although the detrimental effect of few virus, such as Zika virus, on the mouse testis has been investigated, the testicular damage only occurred in mice lacking interferon signaling.^31, 32, 33^ These studies indicated that the antiviral system in mice inhibits virus-mediated testicular damage. Therefore, whether MuV infection permanently disrupts spermatogenesis in mice lacking interferon signaling is worthy of determination.

CXCL10 expression can be upregulated by bacterial and parasitic infections.^34, 35, 36^ In particular, various viral infections, such as rhinovirus, respiratory syncytial virus, hepatitis virus and Ebola virus, induce CXCL10 expression.^37, 38, 39, 40^ These studies suggested that CXCL10 might be involved in the pathogenesis of different infectious diseases. CXCL10 facilitates the recruitment of CXCR3-positive immune cells, including macrophages, dendritic cells and activated T lymphocytes, to infected sites, which has an important role in initiating inflammatory responses against the invading microbial pathogens.^41, 42^ Moreover, several studies have shown that CXCL10 upregulation induces cell apoptosis in certain viral infectious diseases. CXCL10 induces neuronal apoptosis in SIV and West Nile virus encephalitis.^18, 19^ In addition, it promotes cancer cell apoptosis in human papillomavirus-associated cervical carcinoma.^43^ We recently demonstrated that MuV significantly induces CXCL10 expression in mouse testicular somatic cells.^12^ The present study showed that MuV-induced CXCL10 in Sertoli cells triggered the apoptosis of male germ cells in a paracrine manner. Whether CXCL10 upregulation facilitates the development of orchitis by recruiting leukocytes to the testes after MuV infection requires clarification *in vivo*. Notably, Zika virus infection induces CXCL10 production in mouse testicular somatic cells and leads to leukocyte infiltration in the testes, resulting in orchitis.^31, 32^ These results suggest that CXCL10 production might be involved in the pathogenesis of the testes after MuV and Zika virus infection.

Understanding the mechanisms by which MuV induces CXCL10 production in the testes would be helpful for the development of therapeutic interventions in MuV orchitis. The present study shows that TNF-*α* induces CXCL10 expression in an autocrine manner in Sertoli cells after MuV infection. This observation corresponds to a previous report, which showed that TNF-*α* upregulates CXCL10 expression in rat Leydig cells.^21^ However, CXCL10 expression was not completely abolished in TNF-*α*^−/−^ cells, suggesting that TNF-*α*-independent mechanisms should be also involved in MuV-induced CXCL10 expression in Sertoli cells. Although IFN-*γ* induces CXCL10 expression,^13^ this mechanism cannot be involved in MuV-induced CXCL10 production, because MuV did not induce IFN-*γ* expression in co-cultures of Sertoli and germ cells (data not shown). Moreover, CXCL10 mRNA was significantly upregulated as early as 6 h after MuV infection, when TNF-*α* was not detected at the protein level. These observations suggested that MuV may also directly induce CXCL10 expression independently of TNF-*α* and IFN-*γ* in Sertoli cells. In support of this speculation, an early study showed that HIV envelope glycoprotein gp120 induces CXCL10 expression independent of IFN-*γ* in the mouse brain.^44^ Whether MuV envelope glycoprotein directly induces CXCL10 expression in Sertoli cells is worthy of clarification.

In addition to Sertoli cells, Leydig cells in the testicular interstitial spaces also produce CXCL10 in response to MuV infection.^12^ Increased CXCL10 levels in the testicular interstitial spaces may facilitate the migration of leukocytes into the testis, which remains to be tested. By contrast, CXCL10 produced by Leydig cells should not induce the apoptosis of germ cells behind the blood–testis barrier. Therefore, MuV-induced CXCL10 production by Sertoli and Leydig cells may play different roles in the pathogenesis within the testis.

In summary, the present study demonstrates that MuV-induced TNF-*α* upregulates CXCL10 expression in Sertoli cells in an autocrine manner, and that CXCL10 induces male germ cell apoptosis though the activation of caspase-3. The results provide novel insights into the mechanism underlying MuV-impaired spermatogenesis. The CXCL10/CXCR3 system in testicular cells might be considered as a therapeutic target for male infertility caused by MuV infection.

## Materials and methods

### Animals

C57BL/6J strain mice were obtained from the Laboratory Animal Center of Peking Union Medical College (Beijing, China). TNF-*α* knockout (TNF-*α*^−/−^) mice (B6/129S6-TNF^tm1GK1^/J) with C57BL/6J background and CXCL10^−/−^ mice (B6.129S4-CXCL10^tm1Adl^/J) with C57BL/6 background were purchased from the Jackson Laboratory (Bar Harbor, Maine, USA). WT control mice were generated by backcrossing knockout mice to C57BL/6J mice. All of the mice were maintained in a pathogen-free facility on a 12 h/12 h light/dark cycle with access to food and water *ad libitum*. All mice were handled in compliance with the Guidelines (permit number: SCXK (Jing) 2007-0001) for the Care and Use of Laboratory Animals established by the Chinese Council on Animal Care (Beijing, China). All experimental procedures were approved by Institutional Animal Care and Use Committee of the Institute of Basic Medical Sciences in China.

### Reagents

Goat polyclonal anti-CXCR3 (sc-9901), rabbit polyclonal anti-LHR (sc-25828) and anti-WT1 (sc-192) antibodies were purchased from Santa Cruz Biotechnology (Santa Cruz, CA, USA). Rabbit polyclonal anti-Caspase-3 (9662S) and mouse monoclonal anti-Caspase-8 (9746S) antibodies were purchased from Cell Signaling Technology (Beverly, MA, USA). Mouse monoclonal anti-MuV nucleoprotein (ab9876) and rabbit polyclonal anti-MVH (ab13840) antibodies were purchased from Abcam (Cambridge, UK). Mouse monoclonal anti-*β*-actin antibody (A5316) was purchased from Sigma (St. Louis, MO, USA). Horseradish-peroxidase (HRP)-conjugated secondary antibodies were purchased from Zhongshan Biotechnology Co. (Beijing, China). Recombinant mouse CXCL10 (250-16) and TNF-*α* (315-01A) were purchased from Peprotech (Rocky Hill, CT, USA). DEVD-fmk (264156), an inhibitor of caspase-3, was purchased from Calbiochem (La Jolla, CA, USA). Pomalidomide (S1567), an inhibitors of TNF-*α*, was purchased from Selleckchem (Houston, TX, USA). Annexin V-FITC apoptosis detection kit (FXP018) and ELISA kit for detecting mouse TNF-*α* (CME0004) were purchased from Beijing 4A Biotech Company (Beijing, China). ELISA kit for detecting mouse CXCL10 (BMS6018) was purchased from eBioscience (San Diego, CA, USA).

### Cell isolation

Testicular cells were isolated from 4-week-old mice based on previously described procedures.^45^ In brief, mice were anesthetized with CO_2_ and then killed by cervical dislocation. The testes were decapsulated and incubated with 0.5 mg/ml collagenase type I (Sigma) in PBS at room temperature for 15 min with gentle oscillation. The suspensions were filtered using 80 *μ*m copper meshes to separate interstitial cells and seminiferous tubules. Interstitial cells were cultured in F12/DMEM (Life Technologies, Grand Island, NY, USA) supplemented with 100 U/ml penicillin, 100 mg/ml streptomycin and 10% fetal calf serum (FCS, Life Technologies). After 24 h, Leydig cells were detached by treatment with 0.125% trypsin for 5 min. Testicular macrophages were not detached in this treatment. The purity of Leydig cells were more than 95% based on staining for LHR (a maker of Leydig cells).^46^ Macrophages in Leydig cell preparations were less than 3% based on the immunostaining for F40/80 (a marker of macrophages).^47^

The seminiferous tubules were recovered and suspended in collagenase type 1 at room temperature for an additional 15 min to remove the peritubular myoid cells. The tubules were cut into small pieces (~1 mm) and incubated with 0.5 mg/ml hyaluronidase (Sigma) at room temperature for 10 min with gentle pipetting to dissociate Sertoli and germ cells. Cell suspensions were cultured in F12/DMEM medium supplemented with 10% FCS at 32 °C for 6 h. Germ cells were recovered by collecting non-adherent cells. The purity of the germ cells was >95% based on immunostaining for MVH, a marker of germ cells.^48^

Sertoli cells were cultured at 37 °C for another 24 h and treated with a hypotonic solution (20 mM Tris, pH 7.4) for 1 min to remove the germ cells that adhered to Sertoli cells. Sertoli cells purity was>95% based on immunostaining for WT1, a marker of Sertoli cells.^49^

### Co-cultures of Sertoli and germ cells

Sertoli cells were seeded in six-well plates at a density of 1 × 10^5^ cells per well. After 24 h, 1 × 10^6^ germ cells were added to Sertoli cells in each well. Twenty four hours later, non-adherent germ cells were removed by washing twice with culture media and the co-cultures were infected with MuV.

### MuV infection

MuV (SP-A strain) was isolated from a mumps patient^50^ and obtained from the Institute of Medical Biology, Chinese Academy of Medical Sciences (Kunming, China). MuV was amplified and titrated in Vero cells. MuV preparations were diluted in 1 × PBS at a density of 1 × 10^9^ PFU/ml and stored at −80 °C. MuV was added to cell cultures at a multiplicity of infection of 5 for *in vitro* infection. For *in vivo* infection, 5-week-old mice were anesthetized with pentobarbital sodium (50 mg/kg) and the testis were surgically exposed. The testis was locally injected with 1 × 10^7^ PFU MuV in 10 *μ*l of PBS using 30-gauge needles. The testis of control mice was injected with an equal volume of PBS.

### Real-time qRT-PCR

Total RNA was extracted using Trizol reagent (Invitrogen, Carlsbad, CA, USA) according to the manufacturer’s instructions. After treatment with RNase-free DNase I (Invitrogen) to remove genomic DNA contamination, RNA (1 *μ*g) was reverse transcribed into cDNA in a volume of 20 *μ*l containing 2.5 *μ*M random hexamers, 2 *μ*M dNTP and 200 U Moloney murine leukemia virus reverse transcriptase (Promega, Madsion, WI, USA). Quantitative PCR was performed in a 20 *μ*l reaction mixture containing 0.2 *μ*l cDNA, 0.5 *μ*M forward and reverse primers, and 10 *μ*l Power SYBR Green PCR Master Mix (Applied Biosystems, Foster City, CA, USA) on an ABI PRISM 7300 real-time cycler (Applied Biosystems). The transcript levels of target genes were determined using the comparative 2^-ΔΔCT^ method as described in the Applied Biosystems User Bulletin No.2 (P/N 4303859). The following specific primer sequences (forward and reverse) were used: for CXCR3, 5′-ACAGCACCTCTCCCTACGAT-3′ and 5′-TGACTCAGTAGCACAGCAGC-3′ and *β*-actin, 5′-GAAATCGTGCGTGACATCAAAG-3′ and 5′-TGTAGTTTCATGGATGCCACAG-3′.

### Western blot analysis

Cells or tissues were lysed using RIPA lysis buffer containing protease inhibitor cocktail (Sigma). The protein concentration was determined using the bicinchonic acid protein assay kit (Applygen Technologies Inc., Beijing, China). The proteins (20 *μ*g/lane) were separated on 10% SDS-PAGE gel and electrotransferred onto PVDF membranes (Millipore, Bedford, MA, USA). The membranes were blocked on Tris-buffered saline (pH 7.4) containing 5% non-fat milk at room temperature for 1 h and incubated with the primary antibodies overnight at 4 °C. The membranes were washed twice with appropriate HRP-conjugated secondary antibodies (Zhongshan Biotechnology Co.) at room temperature for 1 h. Antigen/antibody complexes were visualized using an enhanced chemiluminescence detection kit (Zhongshan Biotechnology Co.).

### Histological analysis and IF staining

For histological analysis, the testis of 5-week-old mice was fixed in 4% paraformaldehyde for 24 h and embedded in paraffin. The paraffin sections (5 *μ*m in thickness) were cut with a rotary microtome Reichert 820 HistoSTAT (Reichert Technologies, Depew, NY, USA). The sections were stained with hematoxylin and eosin for histological analysis on spermatogenesis.

For IF staining, the slides were soaked in citrate buffer and then heated in a microwave at 100 °C for 10 min to retrieve the antigens or the cells were fixed with pre-cooled methanol for 3 min and then permeabilized with 0.2% Triton X-100 in PBS for 15 min. After blocking with 5% preimmune goat sera in PBS for 1 h at room temperature, the sections were incubated with primary antibodies overnight at 4 °C. After washing twice with PBS, the sections were incubated with FITC- or TRITC-conjugated secondary antibodies at room temperature for 1 h. The slides were mounted by antifade mounting medium with DAPI (Zhongshan Biotechnology Co.). Negative controls were incubated with preimmune animal serum instead of the primary antibodies. The sections were counterstained with hematoxylin and mounted with neutral balsam (Zhongshan Biotechnology Co.).

### Enzyme-linked immunosorbent assay

Cells were cultured in six-well plates. Culture media were collected at 24 h after MuV infection. The testis was lysed by grinding in 1 × PBS and the supernatants of the lysates were collected after centrifugation at 1000 × *g* for 5 min. TNF-*α* and CXCL10 levels were measured using ELISA kits in accordance with the manufacturer’s instructions.

### Flow cytometry

Male germ cells were washed twice in 1 × PBS and labeled with Annexin V-FITC using apoptosis detection kit (FXP018, Beijing 4A Biotech Company) following the manufacturer’s instructions. The cells were analyzed with a BD Accuri C6 flow cytometer (BD Biosciences, Franklin lakes, NJ, USA).

### AO/EB staining

Testicular cells were cultured in six-well plates with 2 ml media. At 24 h after MuV infection, 2 *μ*l fluorescent staining solution containing 100 *μ*g/ml AO and 100 *μ*g/ml EB (Sigma) was added to each well and incubated for 1 min at room temperature. Cells were observed under a fluorescent microscope (BX51, Olympus, Tokyo, Japan). Apoptotic cells were shown as ‘orange’ and living cells as ‘green’.

### Terminal deoxynucleotidyl transferase-mediated dUTP nick end labeling

Paraffin sections of the testis were prepared for analyzing germ cell apoptosis. Apoptotic germ cells *in situs* were detected using a TUNEL kit (Maibio Biotechnology Co., Shanghai, China) according to the manufacturer’s instruction. The sections were stained by IF staining with antibodies to MVH, a germ cell marker.

### Statistical analysis

All data are presented as the mean±S.E.M. of at least three independent experiments. Statistical significance between individual comparisons was determined by Student’s *t*-test. One-way ANOVA with Bonferroni’s (selected pairs) post hoc test was used for multiple comparisons. The calculations were performed using SPSS Version 13.0 (SPSS Inc., Chicago, IL, USA) and *P*<0.05 was considered statistically significant.

## Publisher’s Note

Springer Nature remains neutral with regard to jurisdictional claims in published maps and institutional affiliations.
