# Correction: C-X-C motif chemokine ligand 10 produced by mouse Sertoli cells in response to mumps virus infection induces male germ cell apoptosis

**DOI:** 10.1038/s41419-018-0834-0

**Published:** 2018-07-16

**Authors:** Qian Jiang, Fei Wang, Lili Shi, Xiang Zhao, Maolei Gong, Weihua Liu, Chengyi Song, Qihan Li, Yongmei Chen, Han Wu, Daishu Han

**Affiliations:** 1 Department of Cell Biology, Institute of Basic Medical Sciences, Chinese Academy of Medical Sciences, School of Basic Medicine, Peking Union Medical College, Beijing, China; 2 Joint International Research Laboratory of Agriculture and Agri-product Safety, Institute of Epigenetics and Epigenomics, College of Animal Science and Technology, Yangzhou University, Yangzhou, China; 3 Institute of Medical Biology, Chinese Academy of Medical Sciences, Kunming, China

Correction to: *Cell Death & Disease* **8**, e3146; 10.1038/cddis.2017.560; published online: 26 Oct 2017.

The PDF and HTML versions of the article have been updated to include the Creative Commons Attribution 4.0 International License information.

